# Mini Review: Advances in 2-Haloacid Dehalogenases

**DOI:** 10.3389/fmicb.2021.758886

**Published:** 2021-10-15

**Authors:** Yayue Wang, Qiao Xiang, Qingfeng Zhou, Jingliang Xu, Dongli Pei

**Affiliations:** ^1^College of Biology and Food, Shangqiu Normal University, Shangqiu, China; ^2^College of Life Sciences, Henan Normal University, Xinxiang, China; ^3^School of Chemical Engineering, Zhengzhou University, Zhengzhou, China; ^4^Zhengzhou Tuoyang Industrial Co., Ltd., Zhengzhou, China

**Keywords:** 2-haloacid dehalogenases, protein structure, catalytic mechanism, biochemical properties, application

## Abstract

The 2-haloacid dehalogenases (EC 3.8.1.X) are industrially important enzymes that catalyze the cleavage of carbon–halogen bonds in 2-haloalkanoic acids, releasing halogen ions and producing corresponding 2-hydroxyl acids. These enzymes are of particular interest in environmental remediation and environmentally friendly synthesis of optically pure chiral compounds due to their ability to degrade a wide range of halogenated compounds with astonishing efficiency for enantiomer resolution. The 2-haloacid dehalogenases have been extensively studied with regard to their biochemical characterization, protein crystal structures, and catalytic mechanisms. This paper comprehensively reviews the source of isolation, classification, protein structures, reaction mechanisms, biochemical properties, and application of 2-haloacid dehalogenases; current trends and avenues for further development have also been included.

## Introduction

Halogenated organic compounds show excellent thermal conductivity, insulation, heat resistance, lipophilicity, and biological activity (Kim et al., 2020; Zakary et al., 2021). They are widely used in industrial, agricultural, medical, and military fields as cleaning agents, biocides, gasoline additives, solvents, degreasers, pesticides, and intermediates for chemical synthesis, yielding enormous economic and social benefits (Kurumbang et al., 2014; Zhang et al., 2019; Gul et al., 2020b; Ameen et al., 2021). However, increasing amounts of halogenated compounds are discharged into the environment due to overproduction and extensive use, which results in environmental contamination. These compounds spread in lakes, drinking water, groundwater, seawater, and soil. Unlike naturally occurring halogenated compounds, which can be used as antibiotics to treat bacterial infections, man-made halogenated compounds, which are used as degreasers, solvents, biocides, pharmaceuticals, cleaning agents, and in many other industrial applications, are dangerous when introduced to the environment (Wu et al., 2019; Kirkinci et al., 2021). This is because these compounds do not degrade easily in natural environments because of their chemical stability, resulting in their environmental accumulation. Moreover, these compounds can become concentrated and accumulate in organisms through the food chain, with carcinogenic, teratogenic, and mutagenic effects (Fan et al., 2020; Lou Y. Y. et al., 2021; Zhang C. et al., 2021). This poses a serious threat to human health and has become an issue of concern all over the world (Artabe et al., 2020; Lou Y. Y. et al., 2021).

As the main decomposers in nature, microorganisms convert complex organic compounds into simple compounds, thus maintaining the cycle of elements that are vital to life (Hellal et al., 2021; Kajla et al., 2021; Yu et al., 2021). Microorganisms growing in environments polluted by organic halogenated compounds have the potential to transform these compounds owing to the presence of enzymes that catalyze dehalogenation in their cells, called dehalogenases (Atashgahi et al., 2018; Oyewusi et al., 2020b, 2021b). Among them, 2-haloacid dehalogenases are a family of critical enzymes that hydrolytically catalyze the dehalogenation of 2-haloacids to form corresponding 2-hydroxy acids (Kurihara and Esaki, 2008; Adamu et al., 2020). They cannot only degrade toxic pollutants with low energy consumption but also have a wide substrate profile and high catalytic efficiency. They have highly chiral resolution properties, which may enable the production of optically pure 2-halogenated and 2-hydroxyl compounds (Oyewusi et al., 2020a). Hence, 2-haloacid dehalogenases are highly valuable in the field of environmental remediation and environmentally friendly manufacturing of chiral chemicals. Here, we review the isolation source, classification, molecular structure, catalytic mechanism, catalytic properties, and industrial applications of 2-haloacid dehalogenases. These will enrich the biocatalytic repertoire of haloacid dehalogenases and broaden their applications and developments in the future.

## Isolation Sources and Classification of 2-Haloacid Dehalogenases

Microorganisms possessing 2-haloacid dehalogenase are widespread in nature, and have been explored since the beginning of the 20th century. So far, increasing numbers of bacterial and fungal species capable of degrading halogenated xenobiotic pollutants have been isolated (Table 1). Most of these microorganisms were isolated from terrestrial environments, with only a few from marine environments, including *Burkholderia* sp. I37C (Chiba et al., 2009), *Rhodobacteraceae* sp. (Novak et al., 2013a), *Psychromonas ingrahamii* (Novak et al., 2013b), *Pseudomonas stutzeri* DEH130 (Zhang et al., 2013), *Paracoccus* sp. DEH99 (Zhang et al., 2014), *Lysinibacillus boronitolerans* MH2 (Heidarrezaei et al., 2020), and *Bacillus megaterium* BHS1 (Wahhab et al., 2020). The marine environment is the primary and optimal sink for halogenated pollutants because of their natural release by marine macroalgae, bacteria, sponges, tunicates, corals, worms, phytoplankton, and other invertebrates (Bidleman et al., 2019). Additionally, marine environments are considered extreme owing to a combination of unique properties including high pressure, high salinity, low temperature, oligotrophy, and special lighting conditions (de Oliveira et al., 2020; Ameen et al., 2021; Zhang J. et al., 2021). Because of this, microorganisms living in this environment are diverse and specific in gene composition and ecological functions; the intracellular enzymes of these microorganisms are correspondingly diverse and specific, conferring physiological and biochemical characteristics such as barophilicity, salt tolerance, cold adaptability, hyperthermostability, chemoselectivity, stereoselectivity, and regioselectivity (Thippeswamy et al., 2021; Zhang J. et al., 2021). The marine environment is therefore expected to be an important source of novel enzymes.

**Table 1:** The reported microorganisms degrading 2-haloalkanoic acids.

**Microorganisms**	**Genus**	**References**
Bacteria	*Agrobacterium*	Köhler et al., 1998
	*Alcaligenes*	Hill et al., 1999
	*Ancylobacter*	Kumar et al., 2016
	*Arthrobacter*	Bagherbaigi et al., 2013
	*Azotobacter*	Diez et al., 1996
	*Bacillus*	Horisaki et al., 2011; Ratnaningsih and Idris, 2018; Oyewusi et al., 2021a
	*Burkholderia*	Edbeib et al., 2020
	*Klebsiella*	Idris Ratnaningsih, 2015
	*Lysinibacillus*	Heidarrezaei et al., 2020
	*Methylobacterium*	Kurihara and Esaki, 2008
	*Mesorhizobium*	Zakary et al., 2021
	*Moraxella*	Kurihara et al., 2000
	*Paracoccidioides*	Satpathy et al., 2015
	*Paracoccus*	Zhang et al., 2014
	*Pseudoalteromonas*	Liao et al., 2015
	*Pseudomonas*	Hasan et al., 1994; Park et al., 2003; Schmidberger et al., 2008; Zhang et al., 2013
	*Psychromonas*	Novak et al., 2013b
	*Pyrococcus*	Arai et al., 2006
	*Rhizobium*	Adamu et al., 2016; Oyewusi et al., 2020b
	*Rhodobacteraceae*	Novak et al., 2013a
	*Serratia*	Rosland Abel et al., 2012
	*Sulfolobus*	Xu et al., 2004
	*Xanthobacter*	van der Ploeg et al., 1991
Fungi	*Beauveria*	Satpathy et al., 2016
	*Botrytis*	Bustillo et al., 2003
	*Candida*	Polnisch et al., 1991
	*Dichomitus*	Muzikár et al., 2011
	*Fusarium*	Li et al., 2011
	*Metarhizium*	Satpathy et al., 2016
	*Phanerochaete*	Wang et al., 2009
	*Pycnoporus*	Muzikár et al., 2011
	*Trichoderma*	Bagherbaigi et al., 2013

The 2-haloacid dehalogenases have been classified according to amino acid sequence conservation and substrate selectivity (Wang et al., 2018; Adamu et al., 2020). These enzymes are classified into four types according to their substrate specificities and product configurations: D-2-haloacid dehalogenase (D-DEX, EC 3.8.1.9), L-2-haloacid dehalogenase (L-DEX, EC 3.8.1.2), configuration-inverting DL-2-haloacid dehalogenase (DL-DEXi, EC 3.8.1.10), and configuration-retaining DL-2-haloacid dehalogenase (DL-DEXr, EC 3.8.1.11) (Zakary et al., 2021). D-DEX catalyzes the dehalogenation of D-2-haloalkanoic acids, whereas L-DEX specifically acts on L-2-haloalkanoic acids. DL-DEXi and DL-DEXr act on both enantiomers of substrates, but yield different product configurations. The 2-haloacid dehalogenases in general are divided into Group I and Group II enzymes according to the amino acid sequence homology; D-DEX and DL-DEX belong to Group I and L-DEX to Group II.

## Structural and Catalytic Characteristics of 2-Haloacid Dehalogenases

The structural diversity of 2-haloacid dehalogenases determines their diversity of function. The different types of 2-haloacid dehalogenases have different structures and catalytic mechanisms; an overview of this is provided in this section.

### L-DEX

#### Structural Characteristics and Catalytic Mechanism

L-DEX specifically acts on L-2-haloalkanoic acids to produce D-2-hydroxyalkanoic acids. These enzymes are widespread in nature and their biochemical characteristics and structures have been studied extensively (Satpathy et al., 2016; Wang et al., 2016; Adamu et al., 2020). So far, the three-dimensional (3D) structures of specific L-DEXs and their substrate complexes have been analyzed, including L-DEX YL from *Pseudomonas* sp. strain YL (Hisano et al., 1996), DhlB from *Xanthobacter autotrophicus* GJ10 (Ridder et al., 1997), PH0459 from *Pyrococcus horikoshii* OT3 (Arai et al., 2006), DehIVa from *Burkholderia cepacia* MBA4 (Schmidberger et al., 2007), DehSft from *Sulfolobus tokodaii* (Rye et al., 2009) and DehRhb from *Rhodobacteraceae* (Novak et al., 2013a).

**Figure 1:**
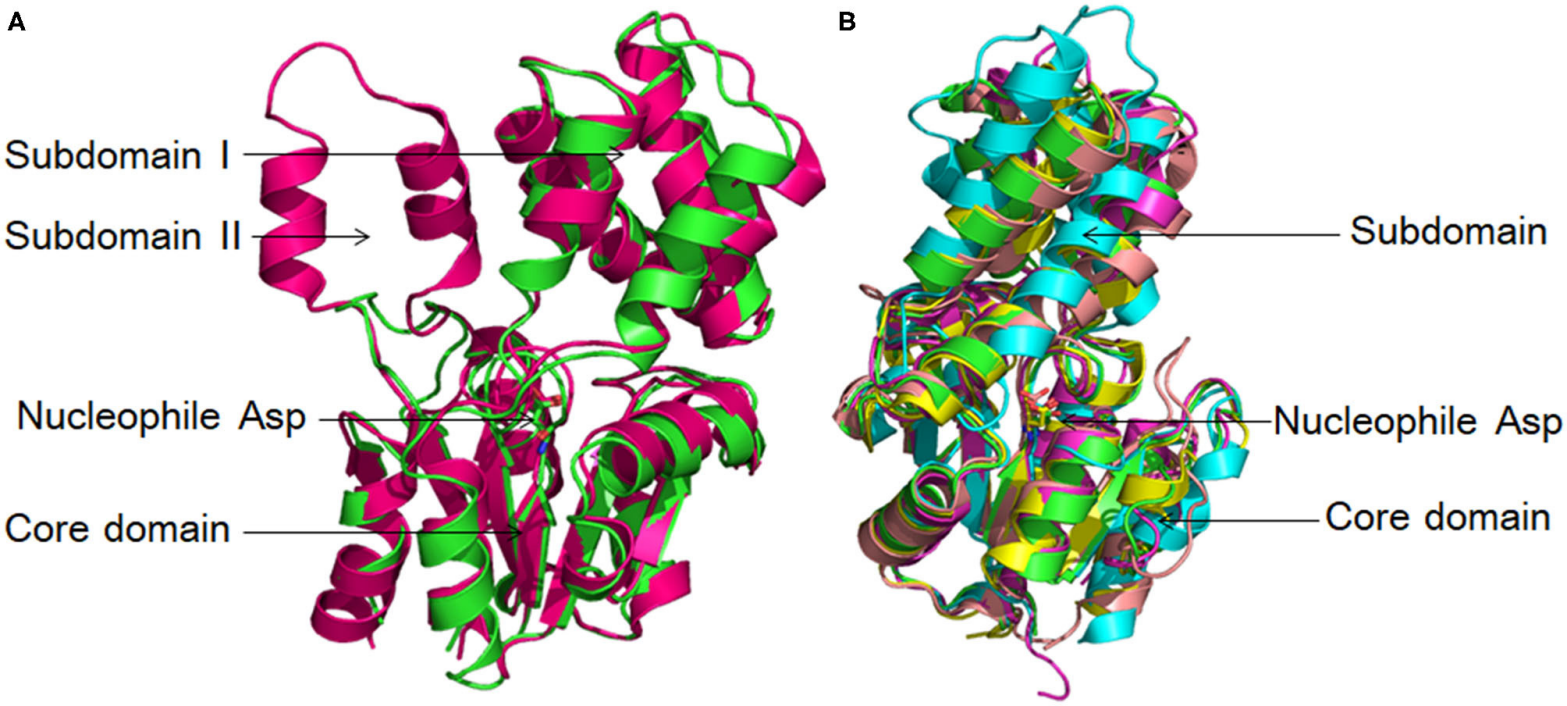
L-DEX structures. **(A)** Structural superposition of DhlB (hotpink, PDB ID: 1qq5) and L-DEX YL (green, PDB ID: 1jud). **(B)** Structural superposition of DhlB (hotpink, PDB ID: 1qq5), L-DEX YL (green, PDB ID: 1jud), PH0459 (cyan, PDB ID: 1x42), DehIVa (magenta, PDB ID: 2no4), DehSft (yellow, PDB ID: 2w11) and DehRhb (salmon, PDB ID: 2yml).

L-DEX is an α/β type hydrolase consisting of a typical Rossman-fold-like core domain and subdomain, with the active site located between the two domains (Figure 1), apart from DhlB, which is composed of a core domain and two subdomains. Most L-DEX molecules are dimers consisting of two identical subunits, except for PH0459, which is a monomer according to its crystal structure (Arai et al., 2006). In a typical L-DEX structure, six-stranded parallel β-sheets (in order: β5-β4-β1-β6-β7-β8) are flanked on both sides by five α-helices, forming three layers of α/β fold units together constituting a sandwich domain (Hisano et al., 1996; Poelarends and Whitman, 2010; Zhang et al., 2018). Although the core domain of L-DEX has an α/β-type structure, it does not belong to the α/β hydrolase fold family, in which the typical domains are eight-stranded β-strands (in order: β1-β2-β4-β3-β5-β6-β7-β8) with the β2-strand antiparallel to the others. Two β-strands are separated by α-helix from the third strand, forming a β/α/β unit. The first α-helix and the last α-helix are located at one side of the β-sheet, and the remaining α-helices are at the other side (Janssen, 2004; Kunka et al., 2018; Babkova et al., 2020; Mazur et al., 2021).

**Figure 2:**
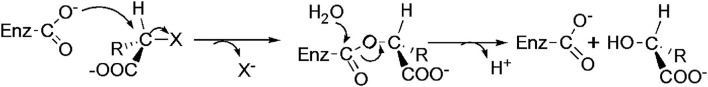
Reaction mechanism of L-DEX (Schmidberger et al., 2007).

The dehalogenation is catalyzed by L-DEX in an S_N_2 nucleophilic substitution reaction as confirmed by X-ray Crystallography, O^18^ isotope labeling, liquid chromatography–mass spectrometry (LC–MS), site-directed mutagenesis, and quantum mechanic/molecular mechanic (QM/MM) calculations (Adamu et al., 2017a,b). The carboxylic acid group of the aspartic acid residue acts as the nucleophile in the active center, which attacks the C2 atom of the L-2-haloalkanoic acid to form an ester intermediate (Schmidberger et al., 2007). This intermediate product is then hydrolyzed by a water molecule, activated by His/Glu (in DehRhb) or Asn/Ser (in DehIVa) or Lys (in L-DEX YL) (Figure 2). The resultant halide ions are stabilized with the assistance of Arg or Asn or Phe. Greater numbers of halide ion acceptors can cleave stronger C-X bonds (Kurihara, 2011; Kondo et al., 2014).

#### Biochemical Properties

L-DEXs have been isolated from both terrestrial and marine environments. Some biochemical characteristics are shared between enzymes, and some differ. For example, L-DEX exhibits high catalytic activity on chlorinated and brominated substrates, but no such activity on D-2-haloalkanoic acids. Additionally, this enzyme cannot catalyze the dehalogenation of fluorinated and *C*_3_-substituted haloalkanoic acids. With the exception of L-DEX YL, L-DEXs only show high catalytic activity on haloalkanoic acids of two or three carbons in length, with low or no activity on haloalkanoic acids four or more carbons in length (van der Ploeg et al., 1991; Liu et al., 1994; Zhang et al., 2013, 2014).

L-DEX enzymes differ in substrate specificity; L-DEX YL is more specific to L-2-chloropropionic acid than chloroacetic acid, whereas the L-DEX from *Bacillus* strain I37C is more specific to chloroacetic acid than to 2-chloropropionic acid (Liu et al., 1994; Chiba et al., 2009). The optimal pH range for L-DEX reactions is 9–11 (alkaline). Subunit molecular weights range from 25 to 28 kDa. Natural L-DEXs exist as monomers, dimers, and tetramers (van der Ploeg et al., 1991; Liu et al., 1994; Zhang et al., 2013, 2014).

L-DEXs isolated from different bacterial species have different thermal stability: the optimum reaction temperature for L-DEX from the terrestrial *Pseudomonas putida* is 30°C−45°C, and it loses 50% activity after 15 min incubation at 55°C. *Psychromonas. ingrahamii* is isolated from the sea-ice interface (−10°C) and exhibits psychrophilic properties; the lowest temperature at which this strain is able to grow is −12°C. L-DEX Pin, from *P. ingrahamii*, has an optimum reaction temperature of 45°C, with a melting temperature of 85°C. L-DEX Pin possesses the characteristics of both psychrophilic and thermophilic enzymes. Structurally, compared with mesophilic enzymes, L-DEX Pin has more hydrophobic surfaces and more salt bridges (Novak et al., 2013b).

The optimum reaction temperature for DehRhb, isolated from marine Rhodobacteraceae, is 55°C. The activity of this enzyme remains at ~45% after incubation for 1 h at 60°C, indicating moderate thermal stability. Its key catalytic residues are His183 and Glu21, which are different from L-DEXs from terrestrial environments, suggesting that it may catalyze the dehalogenation with a novel catalytic mechanism (Novak and Littlechild, 2013). In summary, natural dehalogenases with novel properties may be more likely to be isolated from marine and other extreme environments; a greater understanding of their structures, catalytic mechanism and catalytic properties may provide theoretical guidance for determining the direct evolution of L-DEXs and other dehalogenases.

### DL-DEX

#### Structural Characteristics and Catalytic Mechanism

DL-DEX enzymes, which include DL-DEXi and DL-DEXr, catalyze the hydrolytic dehalogenation of both enantiomers of 2-haloalkanoic acids to produce corresponding 2-hydroxyalkanoic acids.

For DL-DEXi, the configuration of the product is opposite to the substrate: the C2 atom of the substrate configuration is inverted during dehalogenation catalyzed by DL-DEXi. Six DL-DEXi enzymes have been reported so far, including DL-DEX YL from *Pseudomonas putida* YL (Hasan et al., 1994; Soda et al., 1996), DL-DEX 113 from *Pseudomonas* sp. 113 (Nardi-Dei et al., 1999; Park et al., 2003), DehI from *Pseudomonas putida* PP3 (Park et al., 2003; Schmidberger et al., 2008), DL-DEX Mb from *Methylobacterium* sp. CPA1 (Siwek et al., 2013), DehE from *Rhizobium* sp. RC1 (Hamid et al., 2011; Zainal Abidin et al., 2019), and DhIIV from *Alcaligenes xylosoxidans* ABIV (Brokamp et al., 1996; Hamid et al., 2011). The crystal structures of DehI and DL-DEX Mb have been studied, revealing that DL-DEXi is an α-helical hydrolase, with no structural homology to L-DEX and other fold superfamilies in the hydrolases (Schmidberger et al., 2008; Siwek et al., 2013).

**Figure 3:**
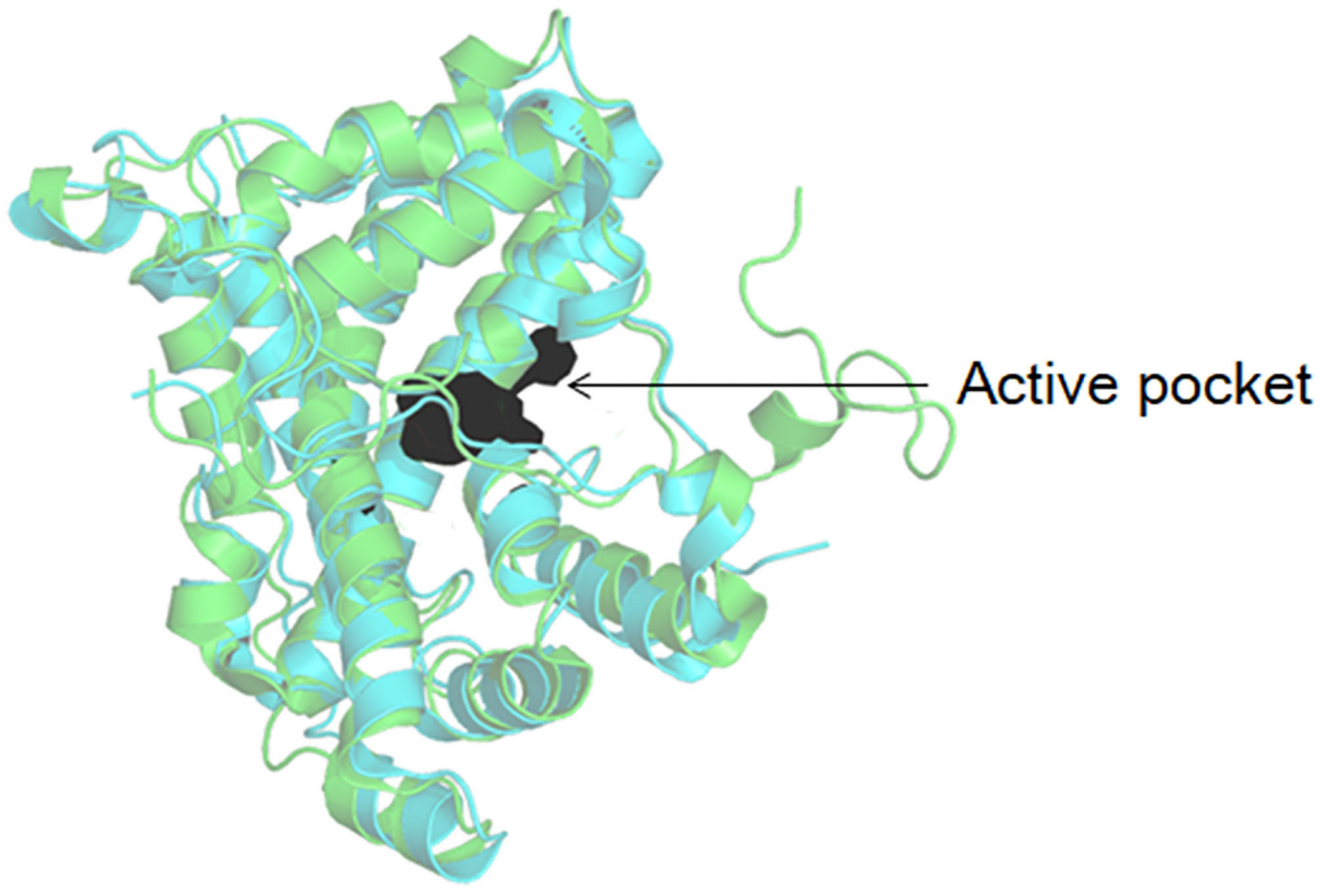
Structural superposition of DehI (green, PDB ID: 3bjx) and DL-DEX Mb (cyan, PDB ID: 4n2x). The active pocket is shown as surface.

**Figure 4:**
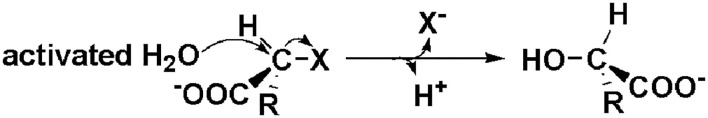
Reaction mechanism of DL-DEXi (Nardi-Dei et al., 1999).

As shown in Figure 3, DehI is a homodimer according to its crystallographic structure. The N-terminus (amino acid residues 1–130) and C-terminus (residues 166–296) share 16% sequence identity in monomers, which form a pseudo-dimer. The active site is located at the interface of the pseudo-dimer, which binds D- and L- substrates (Schmidberger et al., 2008). The catalytic mechanism of DL-DEXi is different to that of L-DEX: dehalogenation catalyzed by D-DEXi is directly mediated by an activated water molecule, without involving the formation of E-S ester intermediate (Figure 4) (Nardi-Dei et al., 1999). The nucleophilic water molecule is likely activated by the conserved Asp and Asn residues; however, there is no relevant experimental evidence for this.

**Figure 5:**
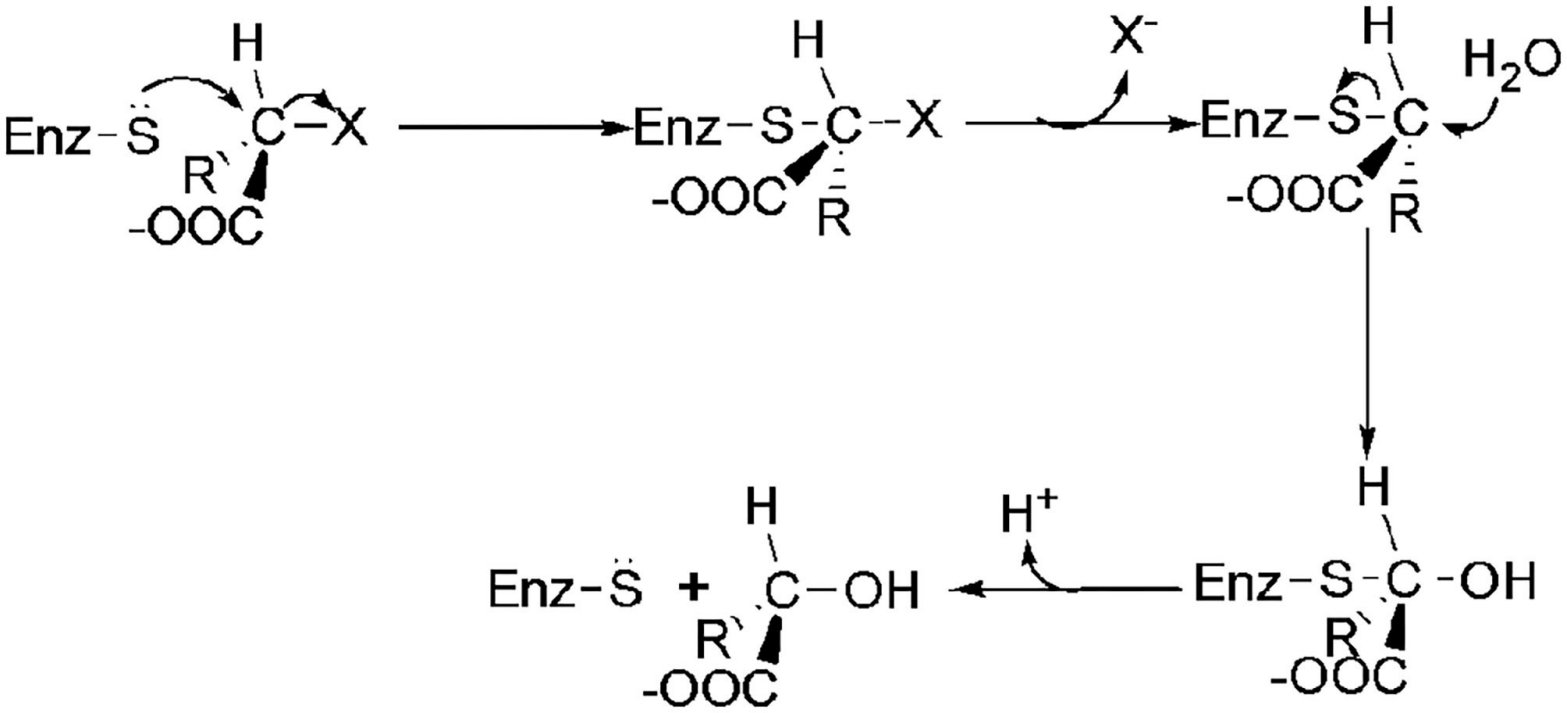
Possible mechanism of DL-DEXr involving the retention of *C*_2_-configuration of the substrate (Weightman et al., 1982).

The transformation of the *C*_2_-configuration of the substrate catalyzed by DL-DEXr is opposite to that of DL-DEXi. DL-DEXr catalyzes dehalogenation with retention of the *C*_2_-configuration of the substrate. Therefore, the substrate and product share the same configuration. DL-DEXr has so far only been reported in *P. putida* PP3 (Weightman et al., 1982; Park et al., 2003). Gene sequence information for this enzyme is still unknown, and the reaction mechanism has not been analyzed. It is proposed that dehalogenation involves a cysteine residue, as DL-DEXr is highly sensitive to sulfhydryl reagents such as *N*-ethylmaleimide and *p*-chloromercuribenzoic acid. The reaction is thought to proceed with double inversion of the *C*_2_-configuration of the substrate, resulting in the retention of the *C*_2_-configuration: the first *C*_2_-configuration inversion releases halogen ions and forms an E-S thioester intermediate; then, the intermediate is hydrolyzed under the attack of a water molecule, and the *C*_2_-configuration is reversed again. However, there is currently no direct experimental data to confirm this hypothesis (Figure 5) (Weightman et al., 1982).

#### Biochemical Properties

In DL-DEXs, only DehI, DehE, DL-DEX 113 and DL-DEX ABIV have been characterized in terms of their enzymatic properties (Brokamp et al., 1996; Schmidberger et al., 2008). These enzymes have a greater specificity for L-2-haloalkanoic acids than D-2-haloalkanoic acids (Table 2). DL-DEXi can catalyze the dehalogenation of haloalkanoic acids with a carbon chain length of two to four, and catalyzes the formation of oxalate from trichloroacetate (Soda et al., 1996). Most DL-DEXi enzymes are homodimers, except for DL-DEX YL, which is a monomer. The subunit molecular weight ranges from 26 to 36 kDa (Kondo et al., 2014). DL-DEXi maximum activity levels occur at a pH of ~9.5. The optimum reaction temperature is between 30 and 40°C (Leigh et al., 1986; Park et al., 2003; Hamid et al., 2011). DL-DEXr is sensitive to SH-reagents; like DL-DEXi, it degrades haloalkanoic acids with a chain length of 2–4 (Weightman et al., 1982).

**Table 2:** Enatioselectivity of DL-DEXis from different strains.

**Enzymes**	**Strains**	**L/D^a^**	**References**
DehI	*P. putida* PP3	1.2	Park et al., 2003
DehE	*R*. sp. RC1	1.6	Hamid et al., 2011
DL-DEX 113	*P*. sp. 113	1.4	Park et al., 2003
DhIIV	*A. xylosoxidans* ABIV	1.1	Brokamp et al., 1996
DL-DEX YL	*P. putida* YL	–	
DL-DEX Mb	*M*. sp. CPA1	–	

a *L/D, the ratio of catalytic activity on L-2-chloropropionic acid and D-2-chloropropionic acid; -, no experimental data is available*.

### D-DEX

#### Structural Characteristics and Catalytic Mechanism

D-DEXs specifically catalyze the hydrolytic dehalogenation of D-2-haloalkanoic acids to produce L-2-hydroxyacids. So far, only four kinds of primary structure information are available for D-DEX, including DehD from *Rhizobium* sp. RC1 (Sudi et al., 2014), DehII from *Agrobacterium* sp. NHG3 (Higgins et al., 2005), HadD AJ1 from *Pseudomonas putida* AJ1 (Smith et al., 1990) and DehDIV-R from *Pseudomonas* sp. ZJU26 (Wang Y. et al., 2020). HadD AJ1 and DehDIV-R share the highest sequence homology (89%); HadD AJ1 and DehII NHG3 share 22.2% sequence homology, and HadD AJ1 and DehD share 32.6% sequence homology.

**Figure 6:**
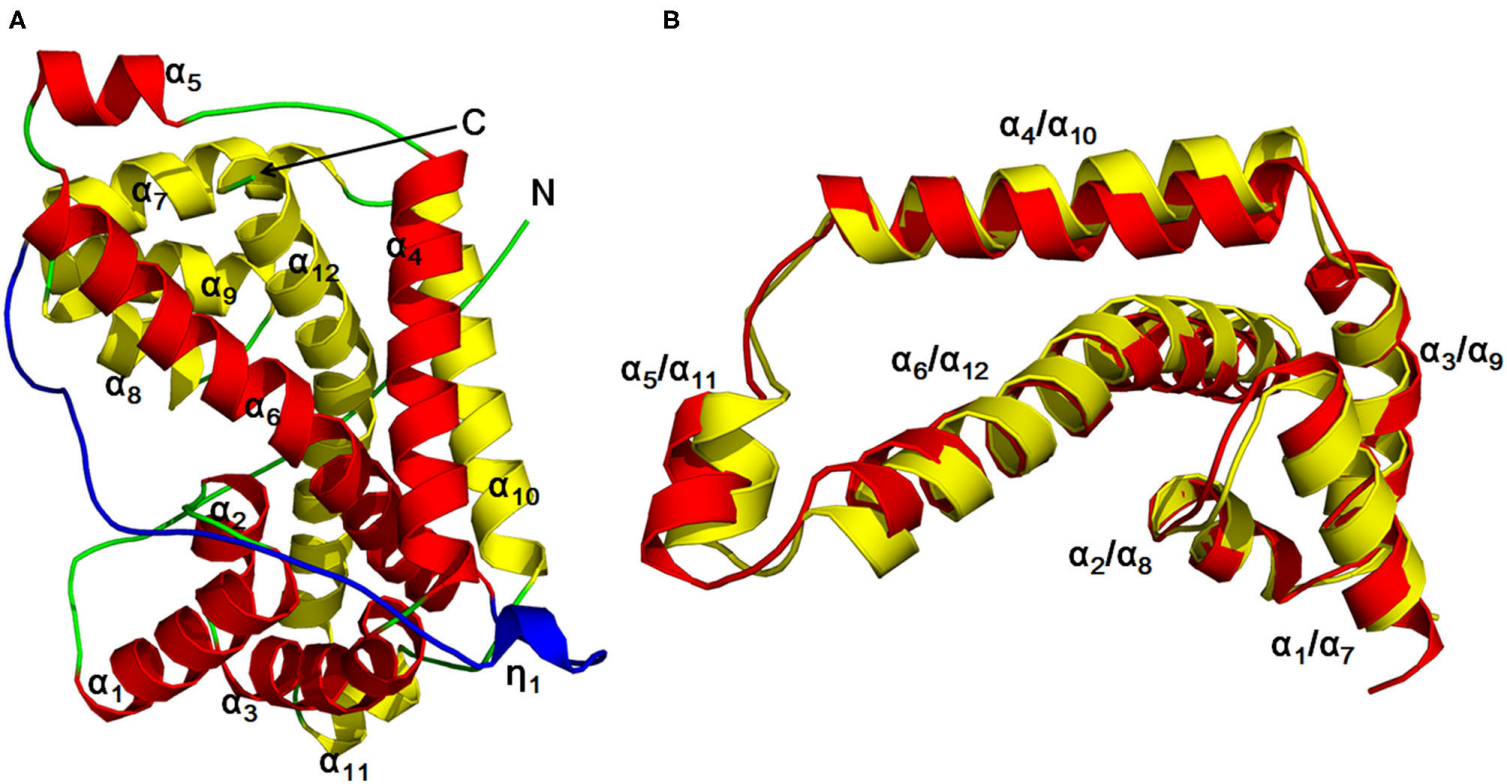
HadD AJ1 monomer (Wang et al., 2018). **(A)** Ribbon representation of monomeric HadD AJ1 composed of two repeats (repeat 1: red; repeat 2: yellow) and a linker (cyan). **(B)** 3D superposition of repeat 1 (red) and repeat 2 (yellow).

The author has extensively studied on the structure and catalytic mechanism of HadD AJ1. The crystal structure of HadD AJ1 is highly similar to that of DL-DEXi. Both types of enzymes are α-helical proteins, different from the α/β fold structure. HadD AJ1 is a homotetramer according to its crystallographic structure; each monomer comprises two repeats with 20% sequence identity (Figure 6). The two repeated folds are composed of N-terminal α-helices 1–6 and C-terminal α-helices 7–12, respectively, with a linker section containing 33 amino acids and a 3_10_-helix η_1_ (Figure 6A). These two repeats are stabilized by van der Waals forces, salt bonds, hydrogen bonds, and hydrophobic interactions. As shown in Figure 6B, helix α_4_ and α_10_ are arranged in parallel with each other, and α_6_ and α_12_ cross each other at the bulge between them. Helices α_6_ and α_12_ mutually interlace at their bulges, located in the middle of the helices (Wang et al., 2018). This has been reported in many proteins with internal structural repeats, which are considered to result from genetic processes such as fusion and fission of domains and gene duplication during protein evolution (Longo et al., 2014; Berezovsky et al., 2017; Vrancken et al., 2020).

**Figure 7:**
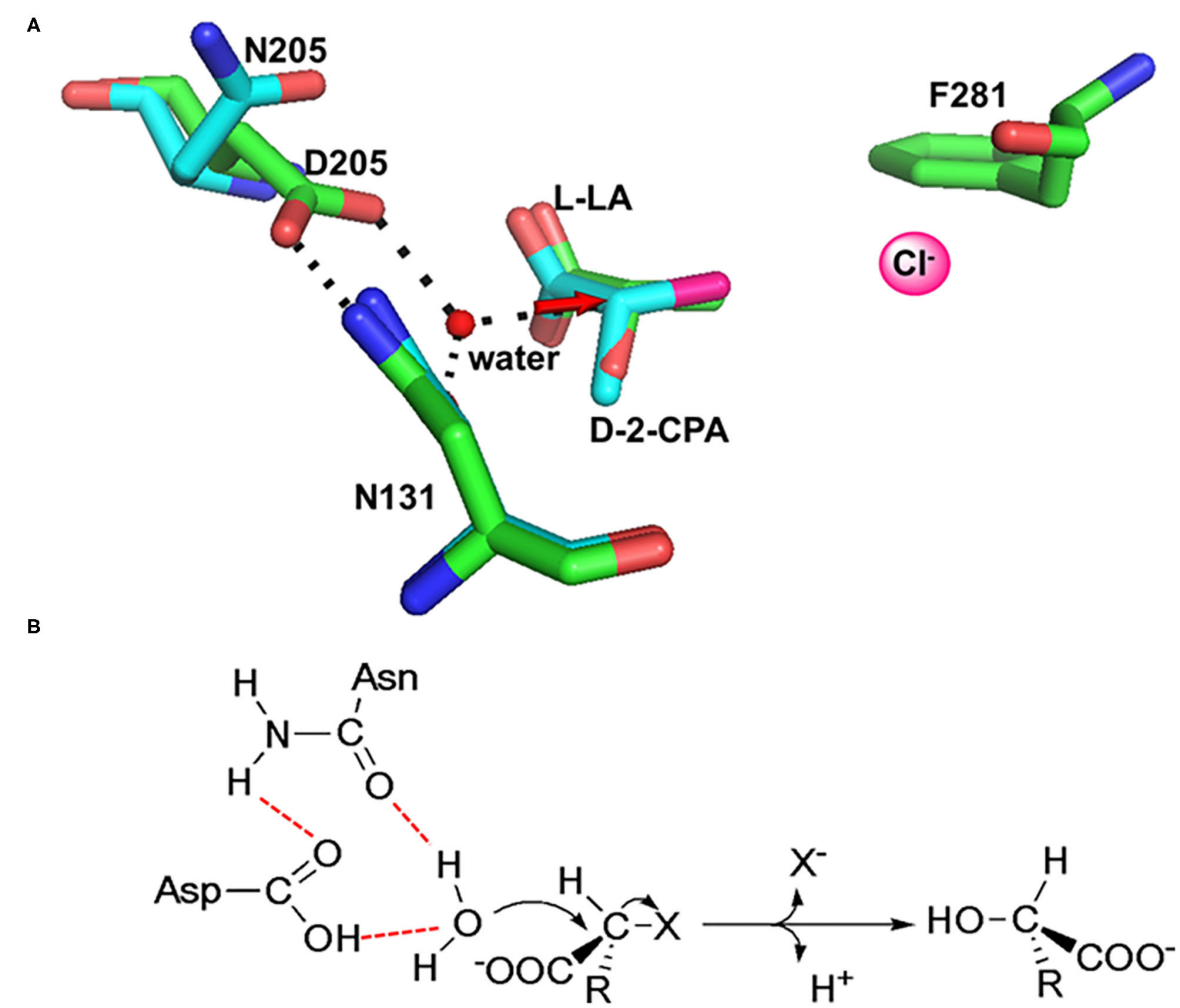
The molecular mechanism of dehalogenation catalyzed by D-DEX. **(A)** Structural superimposition of WT/L-LA (green, PDB ID: 5gzy) and D205N/D-2-CPA (cyan, PDB ID: 5gzx) complex. **(B)** Reaction mechanism of D-DEX (Wang et al., 2018).

In HadD AJ1, Asp205 is the key catalytic residue, activating the water molecule with the assistance of Asn131. This was identified through an analysis of the complex structure of wildtype (WT) enzyme binding the product L-lactic acid (L-LA) and a D205N mutant binding the substrate D-2-chloropropionate (D-2-CPA) (Figure 7A). The dehalogenation catalyzed by D-DEX is directly mediated by activated water molecules, without forming an ester intermediate in the reaction process; this is the same process as DL-DEXi (Figure 7B). The activated water molecule attacks the C2 atom of the substrate from the opposite side of the halogen atom, breaking the C-X bond (Figure 7A). The halogen ion is released toward F281, and simultaneously, the hydroxyl group of the activated water molecule is bonded to the C2 atom of the substrate to form L-lactic acid (Wang et al., 2018).

D-DEX and DL-DEXi share high amino acid sequence as well as structural homology. Moreover, both types of enzymes catalyze dehalogenation by the same mechanism, directly mediated by the nucleophilic water molecule; this differs from dehalogenation catalyzed by L-DEX, which is mediated by E-S ester intermediates. This suggests an evolutionarily close relationship between D-DEX and DL-DEXi.

#### Biochemical Properties

Currently, there are only a few studies on D-DEX enzymes, likely a result of the lack of microorganisms known to produce D-DEX. From analyses of DehD and HadD AJ1 biochemical properties, D-DEXs specifically catalyze dehalogenation of D-2-chlorinated and D-2-brominated acids with carbon chain lengths of 2–4. However, D-DEX has a higher catalytic activity on brominated than chlorinated substrates (Smith et al., 1990; Huyop and Sudi, 2012). *K*_m_ values of DehD, HadD AJ1, and DehDIV-R are 0.06, 0.94, and 2.2 mmol/L, respectively, with D-2-CPA as the substrate (Smith et al., 1990; Huyop and Sudi, 2012; Wang Y. et al., 2020). Compared with HadD AJ1 and DehDIV-R, DehD has a stronger affinity for D-2-CPA.

The natural active states of D-DEXs are different: DehD exists is a homodimer, while HadD AJ1 is a homotetramer. The optimal reaction pH of D-DEXs ranges from 9.0 to 10.0. The enzyme activity decreases rapidly when the pH falls outside the range of 8.0–10.0; under these conditions, HadD AJ1 exhibits <50% catalytic activity (Smith et al., 1990). In comparison with L-DEXs, D-DEXs are mesophilic, with an optimal reaction temperature of 50°C−60°C; however, the enzyme molecules are relatively stable between 30 and 40°C, but rapidly lose activity in a reaction temperature higher than 40°C (Smith et al., 1990).

## Application

The 2-haloacid dehalogenases can detoxify halogenated pollutants by hydrolysis without the addition of other reductive agents; for this reason, their potential application in bioremediation is particularly attractive (Behbahani et al., 2018; Oyewusi et al., 2020b, 2021b; Zakary et al., 2021). The 2-haloacid dehalogenases are also highly stereoselective, and they may therefore be valuable in fine chemistry synthesis applications (Chen and Ribeiro de Souza, 2019; Adamu et al., 2020; Wang S. et al., 2020). These enzymes can be used to obtain chiral hydroxy acids and haloalkanoic acids with low molecular weights; these small organic acids generally act as intermediates for synthesizing agrochemicals, medicines, and other important chemicals (Leemans Martin et al., 2020; Gurushankara, 2021). Hence, 2-haloacid dehalogenases are promising and potentially highly valuable for their application in environmental remediation and chemical synthesis (Bommarius, 2015; Tanokura et al., 2015; Zhang et al., 2018); here, we discuss the main fields in which they could be applied.

### Environmental Bioremediation

Halogenated carboxylic acids such as 2-chloropropionic acids and 2,2-chloropropionic acids are widely used as an intermediate in the synthesis of pesticides and pharmaceuticals, especially the chirally pure 2-chloropropionic acid precursors for synthesizing many chiral drugs (Nguyen et al., 2021; Zhou et al., 2021). However, these haloacids produce chlorinated organic contaminants owing to extensive use and improper disposal. Haloacids are also intermediates in the degradation of some halogenated compounds, such as 1,2-dichloroalkane and hexachlorocyclohexane, which results in more haloacid contaminants in the environment (Hermon et al., 2018). The accumulation of these pollutants causes serious environmental problems and threats to human and other organisms' health. The 2-haloacid dehalogenase can catalyze the dehalogenation of 2-chloropropionic acids and 2,2-dichloropropionic acids to form non-toxic hydroxyl acids, which is a very promising potential tool for environmental bioremediation (Oyewusi et al., 2021b; Zakary et al., 2021). Dioxin compounds are carcinogenic byproducts originating from natural and anthropogenic sources such as herbicides, pesticides, and combustion processes; high levels of dioxin-contamination have been reported in food, soils, and blood samples of local residents in Southern Vietnam (Nguyen et al., 2021). *Burkholderia cenocepacia* strain 869T2 can degrade 0.2 mg L^−1^ of dioxin within 1 week under aerobic conditions, in which L-2-haloacid dehalogenase plays a crucial role (Nguyen et al., 2021). Haloacetic acids are the second most prominent class of disinfection by-products, and are frequently detected in surface and drinking water systems. These compounds have genotoxic, mutagenic, cytotoxic, and tumorigenic effects in humans (Kim et al., 2020; Long et al., 2021; Lou J. et al., 2021). In metabolically engineered *Burkholderia* species, the degradation activity of haloacetic acids can be increased by 4–8 times (Su et al., 2013). The bacterial degradation of haloacetic acids was found to be affected by water distribution system conditions, including pH, phosphate, total organic carbon and residual chlorine (Behbahani et al., 2018). The order of mean haloacetic acid degradation rates has been found to be di >mono >tri-halogenated acetic acids.

Phytoremediation has been attracting more attention as an environmentally friendly technology to clean up environmental contamination (Kurade et al., 2021); transgenic tobacco that produces haloalkane dehalogenase and haloacid dehalogenase, and which therefore contains a complete degradation pathway, has been reported to degrade 1,2-dichloroethane (Mena-Benitez et al., 2008).

### Fine Chemical Synthesis

The growing interest in the use of 2-haloacid dehalogenases in fine chemical synthesis is due to their chiral selectivity. Optically pure compounds are generally synthesized using chemical methods; however, this is unpopular owing to the involvement of toxic reagents, as well as the low yield and low optical purity of products (Santi et al., 2021). Biocatalysis is considered as a more environmentally friendly and effective method because of the mild reaction conditions, and remarkable enantioselectivity (Novak et al., 2013b; Schober and Faber, 2013; Wang S. et al., 2020).

L-2-chloropropionic acid is an important precursor in the synthesis of herbicides and pesticides (Zhou et al., 2021). D-DEX specifically hydrolyzes D-2-chloropropionic acid in racemic 2-chloropropionic acid; L-2-chloropropane acid is therefore obtained with high enantiomeric purity by separation (Gong et al., 2018). To obtain optically active L-2-chloropropionic acid, Imperial Chemical Industries has already applied HadD AJ1 to the resolution of racemic 2-chloropropionic acid in an industrial setting, which has been the primary method for producing chiral chloropropionic acid (Taylor Stephen, 1985; Parker and Colby, 1995). It has also been used by AstraZeneca in the resolution of *rac*-2-CPA by D-DEX. This method is also suitable for the production of other short-chain chiral 2-halogenated acids, and the scale can be higher than 1,000 tons/year (Schober and Faber, 2013).

D-2-CPA is an important raw material for chemical synthesis that can be directly used to produce a variety of pharmaceutical intermediates, such as the nutritional medicine L-alanyl-L-glutamine and the anti-tuberculosis drug thiolactomycin. L-DEX can be used for the resolution of racemic 2-chloropropionic acids to obtain D-2-CPA with enantiomeric purity (Breuer et al., 2004).

Optically pure lactic acid is an important chiral intermediate in the synthesis of agrochemical, pharmaceutical, and chemical industries; it has been reported that L-lactic acid can be used to synthesize nanoparticles and nanofibers, which act as drug carriers (Chuan et al., 2020; Liu et al., 2021; Ma et al., 2021; Yavari Maroufi et al., 2021). D-lactic acid is also involved in the synthesis of important chiral drug intermediates, such as methyl D-lactate (Sengupta et al., 2020). Xie and colleagues studied the reaction conditions of L-DEX from thermophilic archaea *Sulfolobus tokodaii* in the catalytic conversion of racemic 2-chloropropionic acid to D-lactic acid (Rye et al., 2009; Xie et al., 2015); after optimizing reaction conditions with regard to substrate, buffer, and enzyme concentration, preparation of D-lactic acid was found to work best with 0.5 mol/L 2-chloropropionic acid.

D-2-bromobutyric acid is used as an intermediate for the synthesis of pharmaceuticals and agrochemicals. The fluoroacetate dehalogenase mutant H155V/W156R/Y219M is reported to catalyze the kinetic resolution of rac-2-bromobutyric acid, producing D-2-bromobutyric acid with an enantiomeric excess of 99.7% (Wang S. et al., 2020).

### Agricultural Production

Herbicides with broad spectrum can effectively remove a variety of weeds, such as monochloroacetic acid, 2-chloropropionic acid, and 2-dichloropropionic acid. However, these herbicides can also damage economically valuable crops, resulting in significant losses in agricultural production. These losses can be avoided by developing herbicide-resistant crops, which requires the introduction of genes encoding dehalogenases into these crops. The *dehD* gene from *Rhizobium* sp. RCI, encoding D-2-haloacid dehalogenase, has been successfully introduced into tobacco as selective tag, constructing a transgenic variant of *Nicotiana benthamiana* with anti-monochloroacetic acid activity (Mohamed et al., 2020). This transgenic, herbicide-resistant tobacco is confirmed to be effective at various development stages, including seed germination and mature leaf stages. The dehalogenase gene is therefore likely to play an important role as a dominant, selectable marker gene for the construction of other crop species resistant to broad-spectrum halogenated compound herbicides.

### Other Fields

Dehalogenases can also be used to construct biosensors for *in situ* detection of organic halogenated pollutants in the environment (Artabe et al., 2020; Gul et al., 2020a,b). By immobilizing halohydrin dehalogenase on a glass fiber membrane, detection limits of 0.06 mmol/L 1,3-dichloro-2-propanol and 0.09 mmol/L 2,3-dibromo-1-propanol have been achieved (Gul et al., 2020b). A detection limit of 1 mg/L dichlorethane has been achieved by immobilizing haloalkane dehalogenase on stacked chitosan films (Shahar et al., 2019a,b).

Dehalogenases act as tags when genetically fused to a protein of interest, termed HaloTag technology (England et al., 2015; Döbber and Pohl, 2017; Erdmann et al., 2019). This technology overcomes the current limitations of traditional protein tagging platforms, as it can be applied to protein isolation and purification, studies of protein synthesis and degradation, analyses of protein function, studies of protein–protein and protein–DNA interactions, and molecular and cellular imaging (Encell et al., 2012; Merrill et al., 2019; Cattoglio et al., 2020; Freitas et al., 2021; Minner-Meinen et al., 2021). Furthermore, novel technologies have been developed for tumor diagnosis and treatment involving the linkage of dehalogenase fused with cancer cell recognition peptides to multifunctional nanoparticles (Garbujo et al., 2020).

## Discussion and Prospects

A variety of 2-haloacid dehalogenases have so far been isolated and identified. Although structural information and catalytic mechanisms for L-DEX, DL-DEXi, and D-DEX have generally been well-understood, very little information on the structure and catalytic mechanism of DL-DEXr is available. Therefore, further study is necessary to understand DL-DEXr.

Enzyme stereoselectivity has been attracting a great deal of attention for asymmetric synthesis and chiral resolution. The 2-haloacid dehalogenases show typical stereoselectivity; however, little is known about the stereoselective mechanism. The enantioselective mechanism of L-DEX has been studied using quantum mechanics/molecular mechanics (QM/MM) and fragment molecular orbital calculation (Kondo et al., 2015; Adamu et al., 2019), which have confirmed that the high activation energy barrier prevents this enzyme from acting on the D-substrate. However, it is still unclear how selectivity of enzymes on chiral substrates is regulated. The stereoselective mechanism of D-DEX has been studied, and enzymatic stereoselectivity was found to be controlled by the residue Leu288, which determines the entry of L-substrate into the active site of the enzyme with steric hindrance. The mutation of residue leucine to isoleucine enables the enzyme to catalyze the dehaologenation of the L-substrate, owing to the different rotation position of Ile288 compared with Leu288. In the mutant enzyme, Ile288 functions as a wing gatekeeper, interacting with the substrate by gate-flipping during dehalogenation, allowing the L-substrate to enter the active site. However, it is still unclear how DL-DEXr and DL-DEXi recognize and interact with chiral substrates. Stereoselective properties make biocatalysts valuable in the preparation of optically pure compounds, which is an important area of environmentally friendly chemistry. An ideal industrial biocatalyst should have both high catalytic activity and specific stereoselectivity; exploring the molecular regulatory mechanisms underlying these properties forms the basis of artificial customization of dehalogenases with these properties. Reactions can be controlled using direct regulation of enzyme selectivity, forming products with high optical purity and unique structures. Further study on the stereoselectivity of 2-haloacid dehalogenase is therefore required in order to successfully manipulate this property.

Most 2-haloacid dehalogenases have a high catalytic activity with short-chain halogenated acid substrates containing fewer than four carbon atoms, while they show weak or no catalytic activity for longer-chain halogenated acids. Additionally, the low tolerance of these enzymes to organic solvents limits the range of their substrate profile. In order to obtain enantiomerically pure chiral products, enzymatic catalysis is sometimes used in enantiomeric resolution by combining with chemical convergence (Clayton et al., 2020). However, the conditions of the enzymatic reaction are incompatible with the high temperature and extreme pH required for chemical hydrolysis in the downstream separation process. Therefore, it remains necessary to identify novel 2-haloacid dehalogenases with unique properties, allowing them to function in these more extreme conditions (Marshall et al., 2021). Marine microorganisms may be the primary source of novel enzymes with extraordinary properties owing to their previously established genetic and biochemical diversity.

The birth of protein engineering technology has opened up a new route for researchers to develop excellent biocatalysts by redesigning natural enzymes (Marshall et al., 2021; Watanabe et al., 2021; Xiong et al., 2021). Many enzyme engineering design strategies have emerged, such as directed evolution, rational, semi-rational, *de novo*, computer-assisted, and artificial intelligence (Bunzel et al., 2021; Narayanan et al., 2021; Tunyasuvunakool et al., 2021; Woolfson, 2021; Wu et al., 2021). These strategies have been used to improve enzyme stability, activity, and selectivity for substrates. However, so far, only L-2-haloacid dehalogenases have been engineered to alter their substrate specificity. The mutation of residue Ser188 to Val in the enzyme DehE enables it to act on 3-chloropropionic acid (Hamid et al., 2015). Recent developments in understanding the structural and catalytic properties of 2-haloacid dehalogenases will also likely enable these enzymes to be more easily modified for commercial uses alongside L-2-haloacid dehalogenases. Given this overall direction of research, an increasing variety of 2-haloacid dehalogenases will likely be modified through protein engineering techniques to improve their properties for biotechnological applications.

## Author Contributions

YW and QX handled the literature collection and literature research. YW prepared the original draft. QZ, JX, and DP modified the manuscript. All authors critically reviewed, contributed to, and approved the final manuscript.

## Funding

This work was supported by the National Natural Science Foundation of China (22078308), Innovation Leadership Program in Sciences and Technologies for Central Plains Talent Plan (214200510009), Key Scientific Research Projects in the Universities of Henan Province (20A180024), Research Project of Shangqiu Normal University (700144), Postdoctoral Science Foundation of Henan Province (50026003), Program for Science and Technology Innovative Research Team in University of Henan Province (21IRTSTHN025), and Innovation Leadership Program in Sciences and Technologies for Zhengzhou Talent Gathering Plan, Henan Academician Workstation for Industrial Technology of Dry Chilli.

## Conflict of Interest

JX is an employee of Zhengzhou Tuoyang Industrial Co., Ltd. The remaining authors declare that the research was conducted in the absence of any commercial or financial relationships that could be construed as a potential conflict of interest.

## Publisher's Note

All claims expressed in this article are solely those of the authors and do not necessarily represent those of their affiliated organizations, or those of the publisher, the editors and the reviewers. Any product that may be evaluated in this article, or claim that may be made by its manufacturer, is not guaranteed or endorsed by the publisher.
